# Cytokinin Perception in Ancient Plants beyond Angiospermae

**DOI:** 10.3390/ijms222313077

**Published:** 2021-12-03

**Authors:** Sergey N. Lomin, Ekaterina M. Savelieva, Dmitry V. Arkhipov, Pavel P. Pashkovskiy, Yulia A. Myakushina, Alexander Heyl, Georgy A. Romanov

**Affiliations:** 1 Timiryazev Institute of Plant Physiology, Russian Academy of Sciences, Botanicheskaya 35, 127276 Moscow, Russia; losn1@yandex.ru (S.N.L.); savelievaek@ya.ru (E.M.S.); hotdogue@yandex.ru (D.V.A.); pashkovskiy.pavel@gmail.com (P.P.P.); yulia-myakushina@yandex.ru (Y.A.M.); 2 Biology Department, Adelphi University, Science 116, 1 South Avenue, Garden City, NY 11530-070, USA; aheyl@adelphi.edu

**Keywords:** cytokinin, receptor, signaling, plant evolution, binding affinity, ligand preference, pH dependence

## Abstract

Cytokinins (CKs) control many plant developmental processes and responses to environmental cues. Although the CK signaling is well understood, we are only beginning to decipher its evolution. Here, we investigated the CK perception apparatus in early-divergent plant species such as bryophyte *Physcomitrium patens*, lycophyte *Selaginella moellendorffii*, and gymnosperm *Picea abies*. Of the eight CHASE-domain containing histidine kinases (CHKs) examined, two CHKs, PpCHK3 and PpCHK4, did not bind CKs. All other CHK receptors showed high-affinity CK binding (*K*_D_ of nM range), with a strong preference for isopentenyladenine over other CK nucleobases in the moss and for *trans*-zeatin over *cis*-zeatin in the gymnosperm. The pH dependences of CK binding for these six CHKs showed a wide range, which may indicate different subcellular localization of these receptors at either the plasma- or endoplasmic reticulum membrane. Thus, the properties of the whole CK perception apparatuses in early-divergent lineages were demonstrated. Data show that during land plant evolution there was a diversification of the ligand specificity of various CHKs, in particular, the rise in preference for *trans*-zeatin over *cis*-zeatin, which indicates a steadily increasing specialization of receptors to various CKs. Finally, this distinct preference of individual receptors to different CK versions culminated in vascular plants, especially angiosperms.

## 1. Introduction

Hormonal regulation of biological processes is an integral part of the plant growth, development and adaptation to the environment. Due to massive genome sequencing, it has become possible to study the evolutionary formation of individual hormonal systems at the molecular level. Research has focused on the cytokinin (CK) hormonal system, with special attention on its hormone perception and signaling machinery. Apart from the model plant Arabidopsis [1,2,3], this machinery is already pretty well characterized in several representatives of angiosperms: *Zea mays* [4,5,6,7], *Oryza sativa* [8,9], *Medicago truncatula* [10,11], *Brassica rapa* [12], *Malus domestica* [13], *Solanum tuberosum* [14,15], *Populus ssp* [16,17]. However, data concerning species of other groups of higher plants, especially of phylogenetically most distant, are rather scarce [18,19,20,21,22].

In plants, an evolutionary advanced version of a two-component system (TCS) is responsible for the CK signaling. This system consists of a transmembrane (TM) receptor—a sensory hybrid histidine kinase (HK), phosphotransmitters (HPts)—small mobile proteins, and response regulators (RRs)—which can be further subdivided into the RR-B, transcription factors, and the RR-A, negative regulators [3,23,24,25,26]. During signaling, a high-energy phosphate is transferred via conserved protein domains alternately along the histidine-aspartate-histidine-aspartate chain termed Multistep Phosphorelay (MSP), from the TM receptor via HPts to the nuclear type-B response regulator (RR-B). Receiving this phosphorylation signal, RR-Bs are activated and bind to promoters of the primary response genes, changing (mainly activating) their transcription. Among early proteins encoded by primary response genes, RRs type-A counteract the phosphorelay, creating the negative feedback loop in CK signaling. Arabidopsis has three CK receptors termed AHK2, AHK3 and (CRE1/)AHK4. Although they are capable of functionally replacing each other, they are not identical in their biochemical properties and physiological roles in the plant [1,2,3,4,5,6,8,27,28,29,30,31,32].

Active natural CKs are currently assumed to be isoprenoid CKs–nucleobases [6,33]. There are several versions of such CKs: *trans*-zeatin (tZ), isopentenyladenine (iP), dihydrozeatin (DZ), and also *cis*-zeatin (cZ) in some plant species. Among Arabidopsis receptors, AHK3 has a high affinity for tZ and DZ at expense of iP, whereas AHK2 and AHK4 strongly bind tZ and iP but not DZ [2,6,28,32]. *AHK4* is expressed predominantly in the root, while *AHK2* and *AHK3* in leaves [32,34,35]. In cereal plants, receptors are as different as their orthologs in Arabidopsis [4,5,8].

CKs can move in a plant both along the xylem from root to shoot and along the phloem from shoot to root [36]. In this circuit, tZ riboside (tZR) predominantly moves upward along the xylem, and iP riboside (iPR) moves in both directions along the phloem [37]. *Trans*-zeatin is synthesized mainly in the root [38] and is essential for shoot growth [39] and transition to adult stage [40]. As for phloem-transported CKs, they have shown to regulate polar auxin transport and maintain vascular pattern in the root meristem [41]. A hypothesis was put forward about plant organ communication based on different forms of CKs, through receptors with different ligand specificity and different organ patterning, where orthologs of AHK2/AHK3 prevail, as a rule, in leaves, while AHK4-orthologs prevail in roots [2,25,28,31,35,37]. Thus, shoot-borne iP-type CKs obviously regulate the vital activity of the root via AHK4 orthologs, while root-borne tZ-type CKs regulate the vital activity of the shoot via AHK3/AHK2-orthologs [2,28,31].

Beyond flowering plants, CK signaling is studied very little. All land plants, in principle, have a complete set of proteins for the CK signal perception and transmission [20,21,42], as described above. However, there are only a few studies describing the properties of CHK receptors in the bryophytes *Physcomitrium* (formerly *Physcomitrella) patens* (PpCHK1-4) and *Marchantia polymorpha* (MpCHK1) [19,21]. All known cytokinins (iP, tZ, cZ, DZ), their precursors and derivatives were found in the moss *P. patens* [18,19,43]. In addition, 6-benzyladenine (BA)-type aromatic CKs were detected. Furthermore, iP was found both outside and inside the cells, whereas tZ mainly inside; the outside-located iP exhibited high physiological activity in increasing bud number [18].

In *Marchantia* bryophyte, cZ was found to be the most abundant CK. Second and third positions are occupied by tZ (two-fold less) and iP (yet three-fold less), respectively [22].

Nothing is known about CKs in the lycophyte *Selaginella moellendorffii*, but in the closely related species *Selaginella kraussiana*, they suppressed dichotomous root branching caused by auxin [44], which may be a prototype for regulation of lateral root formation. In the fern *Azolla filiculoides*, CKs stimulated the growth of the root apical meristem, while auxins suppressed it [45], which is not typical for this hormone pair as compared to other plant groups.

As for gymnosperms, the bulk of studies of their hormonal systems has been carried out on conifers, where both iP- and Z-type CKs were found [46,47]. In the buds of *Pinus radiata*, active CKs–nucleobases, belonging to the group of isoprenoid CKs, were detected. High- (tZ and iP) and low-activity (DZ and cZ) CKs were present in comparable amounts. No aromatic CKs have been reported [48]. Derivatives of these CKs in the form of ribosides, ribotides and glucosides have also been identified. When applied exogenously, BA was able to hydroxylate to *meta*-, *ortho*-, and *para*-topolins. Such native aromatic CKs were earlier detected in poplars [49,50].

CKs are essential for conifers growing and propagating, they are used to regenerate these plants in in vitro culture [51,52]. The function of CKs in the secondary thickening of coniferous shoots is of great interest. Although in conifers auxin is considered leading hormone in shoot secondary growth [53,54], CKs play an important role too by enhancing auxin effect [55,56]. CKs were detected in the wood and especially in the bark of *Pinus sylvestris*. In the latter, auxin–CK crosstalk was shown recently to be sufficient to determine the wood formation dynamics, where auxin controls cell enlargement rates while CKs drive cell division and auxin polar transport [57].

To our knowledge, no data on the biochemical properties of the CHK receptors from lycophytes or gymnosperms were reported so far. In our work, we tried to fill the gap in our knowledge on the CK perception apparatus of land plants, with special attention to early-divergent species (bryophytes, lycophytes and gymnosperms).

## 2. Results

### 2.1. Ancient CHK Receptors in Comparative and Evolutionary Aspects

**Figure 1:**
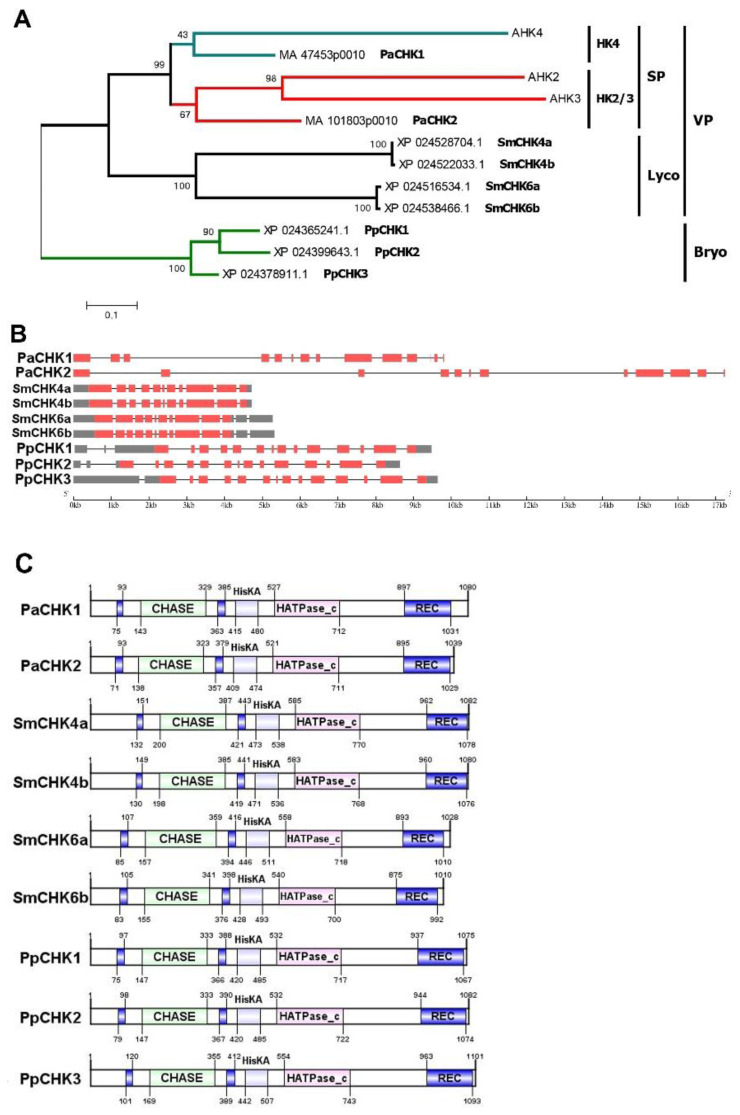
Bioinformatic analysis of the CHKs used in this study. (**A**) Molecular phylogenetic analysis by Maximum Likelihood method. VP—vascular plant, Bryo—Bryophyta, Lyco—Lycophyta, SP—Spermatophyta, HK4—Arabidopsis AHK4 clade, HK2/3—Arabidopsis AHK2 and AHK3 clades. (**B**) CHK gene structures. UTRs are marked gray, ORF are marked pink, bottom scale demonstrates gene length in kilobases. (**C**) CHK protein structures. CHASE domains are flanked by transmembrane domains colored in blue. Positions of domains termini and protein lengths are indicated in amino acid (aa) numbers.

CHK receptors typically consist of three basic modules. The *N*-terminal extracytosolic sensory module is located on one side of the membrane, while catalytic and receiver modules are located in the cytosol, on the opposite side of the same membrane [14,26,58,59]. The sensory module contains the well-known CHASE domain (PF03924) [60,61] which is composed of PAS and PAS-like subdomains. The PAS subdomain is of special importance since it specifically binds the ligand that triggers the MSP. The adequate functioning of PAS subdomain is assured by highly conserved long pivotal α-helix and short α-helix located upstream and downstream of the CHASE domain, respectively [58]. The crystal structure of the sensory module of AHK4 became available since 2011 [33]. At present, CHASE domain is considered a hallmark of CHK receptors among various sensor HKs in plants [62]. Our analysis of genomes of non-flowering land plants: moss *Physcomitrium patens,* lycophyte *Selaginella moellendorfii* and spruce *Picea abies*, uncovered three, two and two typical CHASE-containing HKs, respectively (Figure 1), in accordance with previous data [20,63]. To note, there is still an uncertainty with the receptor number in the lycophyte, in which four CHK-encoding genes were annotated in the GenBank, but they are so similar pairwise (termed as variants “a” and “b” here) (Figure S2) that they seem to represent sequencing repeats of two genuine CHK receptor genes.

Phylogenetically, CHK receptors were divided into a number of distinct clades (groups) (Figure 1A). Receptors of *P. patens* form a separate clade (Bryo, Bryophyta), the rest of CHKs form a common clade of vascular plants (VP) [2,58,63]. This common clade, in turn, is subdivided into the Lycophyta (Lyco) group with *S. moellendorffii* receptors and the seed plant group (SP). The latter group can be further divided into subgroups HK4 and HK2/3. The conifer receptors PaCHK1 and PaCHK2 belong just to these two subgroups, respectively.

Regarding the primary structure of receptor genes, some regularities can be traced (Figure 1B). In vascular plants, receptor CDS are usually composed of 11 exons. However, in some species there are deviations from this general rule. Spruce receptors have two additional small exons at the 3’end, whereas in SmCHK6a receptor of the lycophyte *S. moellendorffii* 2nd and 3rd exons are fused. In the moss *P. patens*, all three CHK receptor genes have a 14-exon structure. The superposition of gene exonic structures on encoded protein structures shows a fairly large similarity of the gene primary structures of all CHK receptors. Consequently, all plant CHK receptors share similar domain structure, and their sensory modules are flanked at both sides by TM helices (Figure 1C). These similar traits are evidence of the common origin of the CHK receptors.

The moss *P. patens* was a unique species to have seven CHK receptor-related proteins, which we designated the sister CHK group (Figure S1). This subfamily was first discovered in phylogenetic studies of bryophytes *P. patens* and *M. polymorpha* [21]. Sister CHKs have severe abnormalities in the sensory module structure [64] (Figure S2). According to crystal structure of AHK4 sensory module [33], this module contains a number of amino acid (aa) residues critical for CK binding [2]. The long pivotal α-helix occupies aa positions 283–327 of the general alignment. The right (distal) side of this α-helix is usually very conserved in *bona fide* CK receptors, but in the sister group, many such positions are substituted by non-conserved residues. Moreover, there is a deletion at the beginning (aa positions 329–334) of the CHASE domain. Three substitutions are particularly noteworthy: Ala, Asp and Gly at positions 360, 434, and 493, respectively. Replacement of conserved Asp434 by different aa (Phe, Tyr, Leu) deserves special attention, since H-bond between Asp434 and adenine moiety of the CK is crucial for the hormone specific binding. In all sister CHKs the conserved Thr at position 450 is missing. In PpCHK6 this site is occupied by Trp, in other CHKs—by Ser. Thus, these CHKs may be considered versions of classical *wol* (*wooden leg*) mutation of Arabidopsis where CK binding is blocked. Collectively, all of the above suggests that sister CHKs bind CKs very weakly if at all. Hence, it is unlikely that these proteins can have a significant impact on CK perception.

On the other hand, to function as hybrid catalytic HKs, sister CHKs have everything they need, primarily HikA-, HATPase-c-, and REC-domains. The first domain (PF00512) contains an H-box with phosphorylatable His, the sequence of HATPase-c-domain (PF02518) includes N-, G1-, F, G2 and G3-boxes. The REC domain (PF00072) has a DD-box, a D1-box with phosphorylatable Asp, and a K-box. All of these consensus regions have a typical conserved structure in all considered proteins (Figure S2). Thus, they seem all to be functional hybrid HKs and their activity can be additive and/or supportive to the signal-transmitting activity of CHK receptors.

### 2.2. Ligand Binding Properties of CHK Receptors from Early-Divergent Plants

We experimentally analyzed the ligand-binding properties of CHK receptors from *P. patens*, *S. moellendorffii*, and *P. abies* using a radiolabeled ligand method and microsomes from tobacco leaves where these receptors were transiently expressed. Tritiated isopentenyladenine served as the labeled CK. PpCHK1 and 2 proteins from *P. patens* were tested in a full-length version, and otherwise sensory modules (SMs) flanked by TM helices were used. PpCHK1-3 represented canonical CHK receptor group, while PpCHK4 was a representative of the related sister group. From each pair of *S. moellendorffii* receptors, probably being two sequencing repeats, one representative (CHK4a-SM and CHK6-SM, in the last case CHK6-SM a and b are identical, so their specification by letter is unnecessary) was taken for further studies. The PaCHK1 and PaCHK2 pair of sensory HKs represents according to current knowledge the entire set of CHK receptors from *P. abies*.

**Figure 2:**
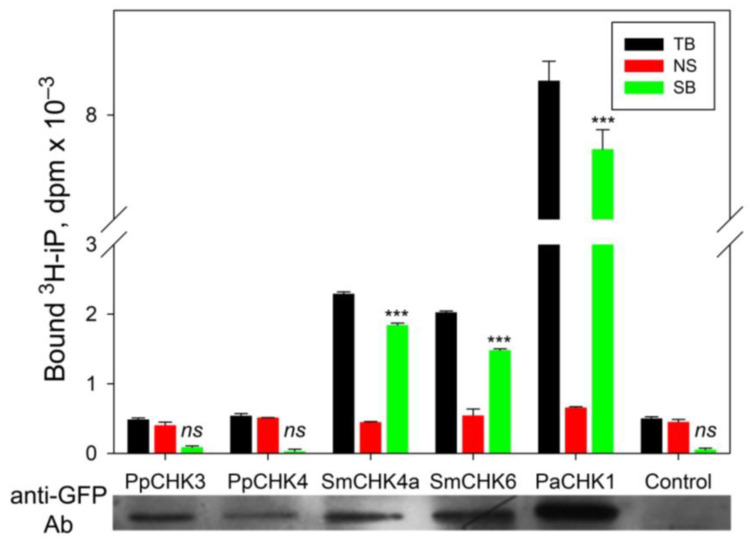
High affinity or lack of labeled iP binding by CHKs from moss *Physcomitrium patens*, lycophyte *Selaginella moellendorffii* and spruce *Picea abies*. For each case total (TB), non-specific (NS) and specific (SB) binding of ^3^H-iP was determined. Control probe shows the level of ^3^H-iP binding by endogenous CK receptors in tobacco leaves. Values for each receptor were validated using one-way ANOVA. Statistical significance of the difference between TB and NS is marked by stars (*** means *p* value ≤ 0.001, low case ns means not significant). At the bottom, Western blot with antibodies (Ab) against GFP protein fused to receptor moiety is shown, serving a proof for the receptor transient expression in tobacco leaves.

Of the eight cloned receptors, we detected no CK binding for PpCHK3 and PpCHK4, although expression of these proteins was confirmed by immunoblotting against the fused GFP domain (Figure 2). The sensory module of the PpCHK3 protein (KJ697770) from *P. patens*, in comparison to the other canonical receptors considered in this work, seems to have on the whole rather conserved structure. The only marked aa substitution is Phe420 instead of conserved Ser. This substitution alone or in combination with others may be responsible for the current (Figure 2) and previous [19] data showing that PpCHK3 lacks the ability to bind labeled iP or tZ, respectively. Thus, from the initially selected eight CHKs, two (PpCHK3 and PpCHK4) were non-functional as the receptors since they did not recognize iP molecules. The remaining six CHKs strongly bound iP and possess typical structure of their sensory modules (Figure S3) indicating that they belong to the genuine CK receptors.

**Figure 3:**
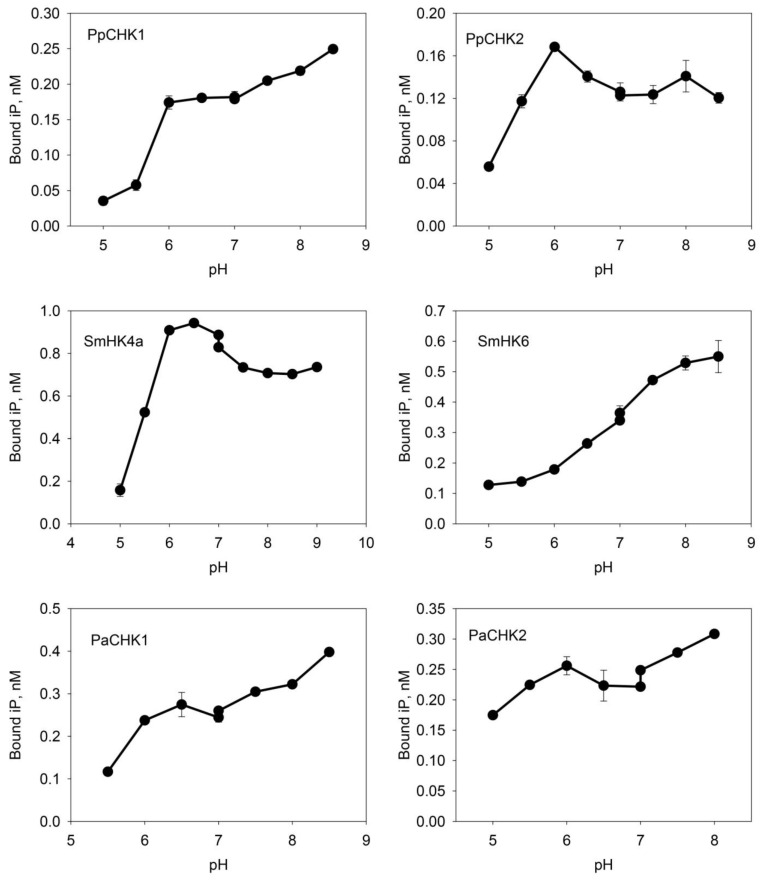
pH-dependences of ^3^H-iP binding by CHK receptors from non-angiosperms: moss *Physcomitrium patens*, lycophyte *Selaginella moellendorffii* and spruce *Picea abies*. Graphics show specific binding (SB). Every curve demonstrates a decrease of CK binding at low pH.

**Figure 4:**
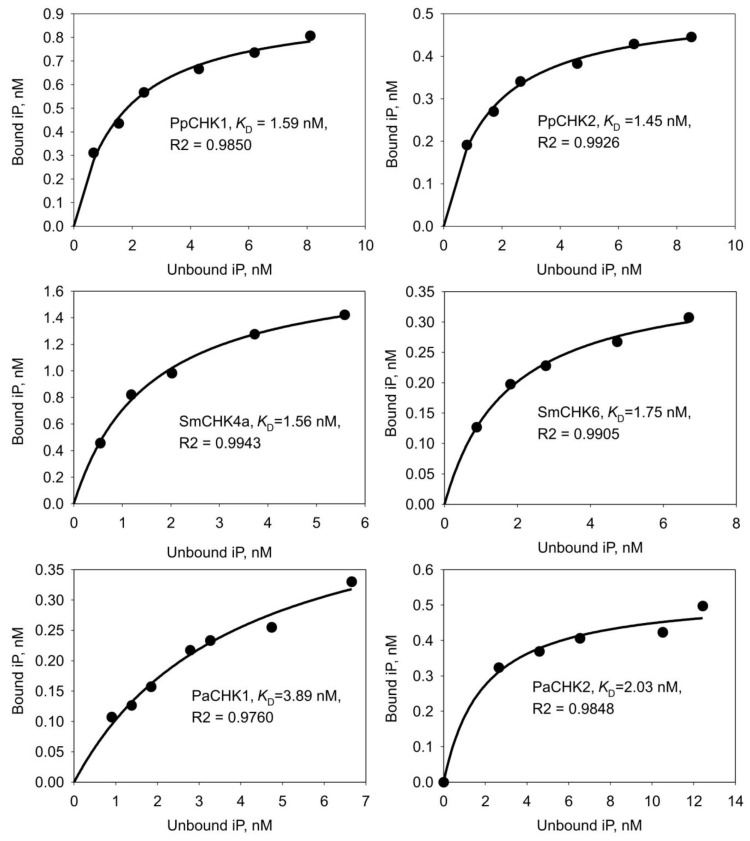
Saturation curves of ^3^H-iP binding to CHK receptors from non-angiosperms: moss *Physcomitrium patens*, lycophyte *Selaginella moellendorffii* and spruce *Picea abies*. Graphics show specific binding (SB). The goodness of fit for the non-linear regression model was estimated using the parameter R2 (see Section 4). The determined *K*_D_ values are very close and fall into narrow range of 1–4 nM, typical for high-affinity hormone-receptor binding.

Then we conducted a detailed study on the pH-dependence of the iP binding to receptors over a wide pH range (from pH 5 to pH 9). All receptors showed a decrease in CK binding at pH below six. Meanwhile the PpCHK1, SmCHK6 and PaCHK1 receptors demonstrated an increase in binding from acidic to the highest alkaline values. In the case of SmCHK6, this increase was almost linear. PaCHK2 showed a similar, though less straight, growth trend. In contrast, PpCHK2 and SmCHK4a had clear binding maxima at pH 6–6.5 (Figure 3). In saturation experiments, the equilibrium dissociation constants (*K*_D_) for iP were determined using the algorithm in the SigmaPlot program. *K*_D_ values ranged from 1.5 to 4.0 nM, which indicated a high affinity of this ligand to the studied CHK receptors (Figure 4).

**Figure 5:**
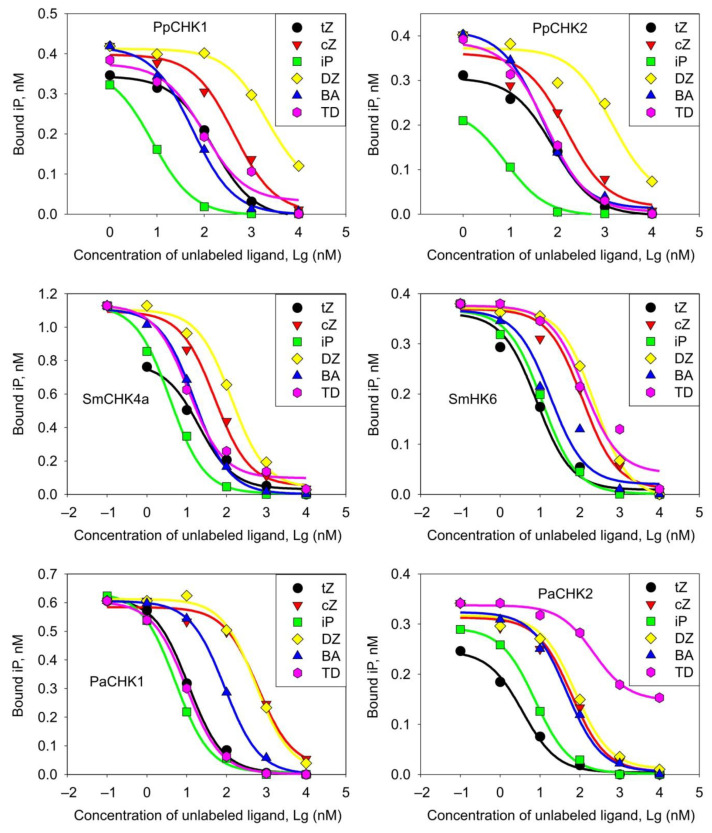
Competition curves of ^3^H-iP displacement by unlabeled CKs from the binding sites in CHK receptors originating from non-angiosperms. tZ, *trans*-zeatin; cZ, *cis*-zeatin; iP, isopentenyladenine; DZ, dihydrozeatin; BA, 6-benzyladenine; TD, thidiazuron.

The specificity of the receptors for six unlabeled hormones: *trans*-zeatin (tZ), *cis*-zeatin (cZ), isopentenyladenine (iP), dihydrozeatin (DZ), benzyladenine (BA), and thidiazuron (TD)—was determined in competition experiments. The dose-response curves of competitive CK binding are shown in Figure 5. Calculation of equilibrium *K*_D_ was carried out by the standard method according to the Cheng-Prusoff formula [65]. The proposed alternative calculation algorithm produced similar results (see Appendix A and Table S2).

The affinity data of various CKs to cognate receptors are shown in Table 1. The *P. patens* receptors exhibited the highest affinity (*K*_D_ < 10 nM) for iP. According to *K*_D_ calculation, affinity for tZ was much lower, while cZ and DZ rounded out the list. In contrast, vascular plant receptors, apart from iP, strongly bound also tZ and BA, but not cZ or DZ. *Selaginella* receptors were distinguished by relatively high affinity towards BA. In addition, SmCHK4a and PaCHK1 strongly bound thidiazuron (TD), a synthetic phenylurea-derived CK. SmCHK6 bound iP and tZ with the close affinities, SmCHK4a and PaCHK1 preferred iP, while PaCHK2 preferred tZ. The highest variation ranges between two CHK receptors of the same species was a feature of spruce receptors. For example, the PaCHK1/CHK2 affinity ratios (*K*_D1_/*K*_D2_) for tZ, cZ, DZ and TD were 7.21, 23.4, 16.8 and 0.1, respectively. For comparison, the corresponding values of PpCHK1/CHK2 affinity ratios for the same CKs: 2.04, 3.01, 1.37 and 1.83, respectively, indicated much less variations in the moss. The maximal to minimal affinity ratio for spruce receptors was equal to quotient of division 23.4/0.1 = 234; the analogous value for moss receptors was only 3.01/1.08 = 2.8. CHK receptors of lycophyte, phylogenetically positioned somewhere between moss and spruce, have an intermediate value of this parameter: 25.7, which is almost an order of magnitude higher than that of *P. patens*, but almost an order of magnitude lower than that of *P. abies*. Both CK receptors from moss hardly distinguished between *cis*- and *trans*-Zeatins, *K*_D_’s for these two CKs for both PpCHK1 and PpCHK2 were very close (Table 1), with the cZ/tZ *K*_D_ ratios being 2.79 and 1.89, respectively. In the lycophyte, at least one CK-receptor already became much more specific. Although SmCHK4a still had a fairly close affinities for cZ and tZ (*K*_D_ ratio 2.58), SmCHK6 recognized tZ much better than cZ (*K*_D_ ratio 10.09). A further enhancement of specificity was detected in spruce CHK receptors: cZ/tZ *K*_D_ ratio for PaCHK1 and PaCHK2 was 55.4 and 17.0, respectively. Thus, during the transition from bryophytes to seed plants, there was a clear development of the receptor preference towards distinct CK versions.

## 3. Discussion

In the work presented here, we studied the main biochemical features of CHK receptors from plants with sequenced genomes belonging to three key groups of land plant evolution [63,66]. These are representatives of bryophytes *Physcomitrium patens*, lycophytes *Selaginella moellendorffii*, and gymnosperms *Picea abies*. The earliest time of their appearance in evolution is estimated as 506.4, 432.5 and 302.8 Ma, respectively (compare with time of Angiospermae appearance—246.6 Ma) [67]. Thus, the selected species are important milestones in the land plant evolution.

### 3.1. Lessons from Experimental Studies of CHK Receptors from Early-Divergent Lineages

#### 3.1.1. Selection of Representative Receptors

We investigated the hormone-binding properties of eight putative CHK receptors from three early-divergent plant species. The receptors of lycophytes and gymnosperms have been studied for the first time at the protein level. Among sensor HKs, PpCHK1 and PpCHK2 of *P. patens* displayed typical properties of CHK receptors, whereas PpCHK3 from the main receptor group and PpCHK4 from a distinct CHK clade termed sister group did not bind CK with receptor-befitting affinity. It was previously noted that PpCHK3 did not bind labeled tZ and that plants with knocked-out *PpCHK1-3* genes stop responding to CK despite the presence of intact genes from the sister group [19]. Our bioinformatic analysis indicated some putative structural reasons for the inability of PpCHK3 and PpCHK4 to bind CKs. All these data cast strong doubt on the real involvement of PpCHK3 and sister CHKs in the perception of the CK signal. These two *P. patens* HKs, which were unable to specifically bind CK, were excluded from further studies. In *S. moellendorffii*, the available four receptor genes are very similar pairwise to each other likely being sequencing repeats of two genuine receptor genes, so we investigated the properties of one receptor of each pair. The ligand-binding properties of both spruce receptors, which we named PaCHK1 and PaCHK2, were also studied in-depth. Thus, for the first time, the ligand-binding properties of the CK perception apparatus of several early-divergent plant species, unique from evolutionary viewpoint, have been experimentally investigated.

#### 3.1.2. The pH Dependence of Ligand Binding

According to presented data, most of the receptors of ancient plants have a typical pH dependence of CK binding, with a decrease at the acidic region, which indicates a preferred intracellular localization. The exceptions were the moss receptor PpCHK2 and the spruce receptor PaCHK2, which retained a sufficiently high ligand-binding activity at pH 5.5 and even pH 5.0 (PaCHK2). This shows the ability of these receptors to function also within the plasma membrane in an apoplastic environment, for which the pH range 4.5–5.5 is typical [68]. Since the main CK in the moss, iP, acts extracellularly [18], it is very likely that it acts principally on the PpCHK2 receptor which resides tentatively in the plasma membrane. Thus, it may be suggested that competent protonema cells—progenitors of bud formation—actively express the *PpCHK2* gene, to be ready to recognize the apparition of extracellular iP, the inducer of bud differentiation.

In turn, the potential localization of most of the CHK receptors inside the cell opens up for them the possibility to perform some basic functions aimed at preserving the intracellular homeostasis. In particular, among putative functions, that of pH sensing seems to be one of most plausible for some reasons. This is especially true for those receptors which are characterized by a monotonic increase in CK binding with increasing pH. Such a quasi-linear pH dependence was observed for receptors from lycophyte SmCHK6, gymnosperms PaCHK1 (Figure 3), as well as from flowering plants: maize ZmHK1 [6], and potato StHK4b [14]. Another reason is the close structural similarity of CHK receptors to bacterial pH sensors. For instance, such a classical pH sensor of bacteria as the chemoreceptor TlpB from *Helicobacter pylori* is a typical TCS HK that perceives a signal, i.e., the level of intracellular pH, in the process of ligand (urea) binding in PAS domain of its sensory module [69]. This is direct analogy with HKs—CHK receptors, which bind their ligands, including urea derivatives, also in the PAS (sub)domain of the sensory module, and in both cases the conserved Asp in the binding site plays the key role in ligand recognition [6].

In the moss *P. patens*, the PpCHK1 receptor exhibits a pH dependence that also resembles a quasi-linear one with increasing ligand binding to the highest pH values (9); this relationship is very different from the pH dependence of another moss receptor, PpCHK2. Therefore, we can assume that the PpCHK1 receptor, which is seemingly located inside the cell, acts also as a pH sensor, maintaining a steady state (neutral—weakly basic pH) of the internal milieu in the cells. Most likely, the CHK receptors are not the only or even the main pH sensors that monitor this parameter of plant cells, but rather act as part of a complex pH-control system.

#### 3.1.3. Ligand Specificity of CHK Receptors

In binding assays, *K*_D_’s of complexes iP–receptors were in the range of 1.5–4.0 nM, which is typical for hormonal receptors in general and CK receptors in particular. The ligand specificity of the receptors was determined in competition experiments. Of all tested CKs, *P. patens* receptors had a clear preference for iP. Receptors of later diverging (or modern) land plants increase their affinity for tZ, but retain high iP binding affinity as well. While one of the two pairs of receptors (SmCHK6) in the lycophyte has high and close affinities for iP and tZ, one of the two spruce receptors PaCHK2 binds tZ significantly stronger than iP. Clear changes in the receptor affinity for other ligands were also revealed. For example, the receptors from *Selaginella* (SmCHKs) showed a high affinity for BA, and receptors from spruce (PaCHK1) and again *Selaginella* SmCHK4a— a high affinity for TD. The increase in the specificity of receptors in relation to structurally similar cZ and tZ in the course of plant evolution is particularly indicative. Although both receptors in the moss prefer tZ over cZ, the difference in affinity for *cis*- and *trans*-zeatin was rather small. In the lycophyte, one of the two receptors (SmCHK6) sharply increased the affinity for tZ, while the affinity for cZ remained low. As regards the spruce receptors, the differentiation between *cis*- and *trans*-zeatin was further enhanced, primarily due to a decrease in the affinity for cZ in PaCHK1. It is worth mentioning that the specialization of receptors with respect to *cis*- and *trans*-zeatin did not stop there, but continued in flowering plants in the same direction, albeit in a receptor-specific manner. An example among Angiospermae CK receptors is AHK3 from *A. thaliana*, whose affinity for tZ exceeds that for cZ (cZ/tZ *K*_D_ ratio) by 376-folds [6]. At the same time, there are CK receptors, for example, the ZmHK1 of maize, which retains archaic features, in particular, an almost equal affinity for *cis*- and *trans*-zeatin. Such a sharp differentiation of CK receptors in the ligand specificity indicates further specialization of their functions in plants.

### 3.2. Non-CK-Binding CHKs Can Participate in CK Signaling

Despite the common origin of the moss CHK receptors and CHKs of the sister group (Figure S1), the cellular function of the latter remains obscure. The inability of these proteins to bind CK with high affinity seemingly precludes their participation in CK signaling. However, this blocking relates to CK perception, but not signal transduction stage. The reason for that is the experimentally proven capacity of CK receptors to form heterodimers in vitro [70] and *in planta* [59,71]. In such heterodimers, one CK sensing moiety can be sufficient to recognize the hormone and trigger the signaling process. Furthermore, apart from CK perception, PpCHKs of the sister group evidently have all the functional domains and some have been shown to possess all activities necessary to transduce CK signal up to phosphotransfer proteins [21]. Thus, PpCHKs of the sister group in *P. patens* can play some supporting role as a factor which supplements and enhances the CK signal transduction. The same rational explanation can be true also for the bryophyte PpCHK3 protein which evidently lost its CK binding ability but nevertheless can take part in CK signaling *in planta* [19]. Generally, the activity of various functional HKs may supplement and/or simulate the activity of CHK receptors regardless the presence of CKs. Therefore, to consider the relevant CK-triggered downstream MSP (Multistep Phosphorelay) signaling, it is necessary to take into account the possible participation in the process not only *bona fide* CHK receptors, but also other hybrid HKs.

### 3.3. Probable Scenario for the Evolution of the CK System

It is conceivable that the essential CK functions in early-divergent lineages were restricted to control some basic cellular parameters such as intracellular pH and/or (in mosses) induction of bud formation on the protonema. In contrast with the vast majority of other plant species, *P. patens* has neither homologs of *IPT* genes for direct CK synthesis, nor homologs of cytochrome P *CYP735A* genes which encode enzymes producing tZ-type CKs from iP-type. Therefore, it seems natural that iP, released upon the decomposition of prenylated tRNA, is sufficient in the moss for these simple physiological purposes. Accordingly, moss receptors strongly prefer iP over other widespread CKs. It is noteworthy that the two functional CK receptors in the moss differ not so much in ligand specificity as in pH dependence of ligand binding. It is possible that the latter difference is due to their distinct subcellular localization: one of the receptors is likely located on the plasma membrane and stimulates the bud formation, while the other resides tentatively in the endoplasmic reticulum and maintains somehow the physiological status of the cell (for example, by controlling the intracellular pH). Obviously, during land plant evolution, the number of CK functions has been constantly increasing, so in vascular plants their role has become much more versatile and important [26].

Thus, in the course of evolution, the CK signaling system gained great importance and multifunctionality, which caused its perfecting and rapid development towards the virtual monopolization of MSP; direct, not through tRNA, CK synthesis, as well as expansion of the receptor specificity with their subsequent specialization to certain CKs in flowering plants. At the same time, the CK system acquired greater reliability, stability and plasticity, as evidences, in particular, its resistance against blocking (knockouting) of one or another of its essential elements [31,72] as well as fine-tune coordination of the CK system with other hormonal systems, primarily auxin [73,74] and ethylene [75] ones, to ensure the optimal development of the plant organism.

## 4. Materials and Methods

### 4.1. Bioinformatics Methods

The protein sequences of Arabidopsis cytokinin receptors were used to search for the corresponding genes in other plant species. For this purpose, online services of protein BLAST in the corresponding databases were used. Genes of *Oryza sativa*, *Amborella tricopoda*, *Selaginella moellendorffii*, *Physcomitrium patens* were found in the NCBI database (https://www.ncbi.nlm.nih.gov/, accessed on 10 September 2021); *Picea abies*—in ConGenIE.org (https://congenie.org, accessed on 10 September 2021); *Ginkgo biloba*—in Medicinal Plant Genomics Resource (http://mpgr.uga.edu/, accessed on 14 May 2021); *Azolla filiculoides*—in Fernbase (https://www.fernbase.org/, accessed on 21 September 2021); *Gnetum montanum* and *Anthoceros agrestis*, in The 1000 plant transcriptomes initiative (1KP) (https://db.cngb.org/onekp/, accessed on 24 September 2021); *Marchantia polymorpha*—in Phytozome 13 (https://phytozome-next.jgi.doe.gov/, accessed on 24 September 2021).

For the functional domain search, a number of various online services were used HMMER (https://www.ebi.ac.uk/Tools/hmmer/search/hmmscan, accessed on 24 September 2021) PROSITE (https://prosite.expasy.org/scanprosite/, accessed on 24 September 2021), SMART **(**http://smart.embl-heidelberg.de/, accessed on 24 September 2021). The TMHMM2 program on the DTU Health Tech Web site (https://services.healthtech.dtu.dk/service.php?TMHMM-2.0, accessed on 24 September 2021) was additionally used for TM domain search. Functional protein sequences were verified by the alignment using ClustalW algorithm in MEGA11 [76,77]. The evolutionary history was inferred by using the Maximum Likelihood method based on the Equal Input model [78]. The tree with the highest log likelihood is shown. The percentage of trees in which the associated taxa clustered together is shown next to the branches. Initial tree(s) for the heuristic search were obtained by applying the Neighbor-Joining method to a matrix of pairwise distances estimated using a JTT model. A discrete Gamma distribution was used to model evolutionary rate differences among sites (2 categories (+*G*, parameter = 0.5877)). The tree is drawn to scale, with branch lengths measured in the number of substitutions per site. The analysis involved 12 amino acid sequences. There were a total of 1355 positions in the final dataset. Evolutionary analyses were conducted in MEGA11. The parameters for phylogenetic tree construction are given in the legend to Figure S1. Pictures of gene structures were drawn by Gene Structure Display Server (http://gsds.cbi.pku.edu.cn/, accessed on 11 March 2021) [79]. Structures of proteins with domains were drawn by Illustrator for Biological Sequences (IBS 1.0).

The molecular modeling of 3D CHK receptor structures was run out in Modeller 9.19 software [80], using automodel class for comparative modeling. For each structure 200 models were built, and the best one was chosen according to the DOPE score [81]. The crystal structure of AHK4 receptor (PDB ID: 3T4L) [33] was employed as a template for modeling. Additional structure prediction was performed using IntFOLD Server (Version 6.0) [82]. Models were energy minimized in UCSF Chimera 1.14 [83] using an AMBER 14SB force field [84] with 300 steps of steepest descent and 300 steps of conjugate gradient.

### 4.2. RNA Isolation from Picea abies Fir-Needles

RNA isolation was performed according to the method of [85] with some modifications [86]. For RNA extraction, the following buffer was used: 200 mM Tris-HCl, pH 8.5, 1.5% Li dodecylsulfate, 300 mM LiCl, 10 mM disodium salt EDTA, 1% sodium deoxycholate, 1% Tergitol Nonidet^®^ (Shell Chemicals, The Hague, The Netherlands) P-40 (NP40) and, just before use, 5 mM thiourea, 1 mM aurintricarboxylic acid, 10 mM dithiothreitol, and 2% polyvinylpyrrolidone were added. Plant tissue (200 mg) was ground in liquid nitrogen and the resulting powder was vigorously shaken with the 1.5 mL extraction buffer. The suspension was snap-frozen in liquid nitrogen. After thawing on ice, the extract was centrifuged at 8000× *g* for 60 min at 4 °C. Then 40 µL of 3.3 M sodium acetate (pH 6.1) and 100 µL 100% ethanol were added to the supernatant, and the mixture was chilled on ice for 10 min to. Polysaccharides were pelleted by centrifugation at 8000× *g* for 30 min at 4 °C. In order to precipitate nucleic acids 100 µL of 3.3 M sodium acetate was added and bring to a full test tube by ice-cold isopropanol, and the suspension was left at −20 °C for 24 h. Nucleic acid were pelleted by centrifugation for 60 min at 3000× *g* at 4 °C, resuspended in 600 µL of TE (10 mM Tris-HCl, pH 8.0, 1 mM EDTA) and 600 µL 5 M NaCl and kept on ice for 30 min with periodic vortex mixing. Then the samples were mixed with 300 µL of 10% cetyltrimethylammonium bromide at room temperature and incubated for 5 min at 65 °C to remove residual polysaccharides. Mixtures were extracted with an equal volume of chloroform/isoamylalcohol (24:1, *v*/*v*), bring to a full test tube by 10 M LiCl, mixed, and kept at 4 °C overnight. RNA was pelleted by centrifugation at 16,000× *g* for 60 min at 4 °C, the pellet was washed in 100% ethanol, dried and dissolved in 20 µL TE buffer on ice.

### 4.3. DNA Constructs

#### 4.3.1. Cloning Receptor cDNA

Synthesis of cDNA was performed with 250 ng total RNA, M-MLV Reverse Transcriptase Kit (Fermentas, Canada) and oligo (dT) 21 primer according to manufacturer’s instructions. The sequences of receptor sensory modules were amplified by means of Phusion High-Fidelity DNA Polymerase (Thermo Scientific, Waltham, MA, USA) and were cloning into plasmid pJET1.2 using CloneJET PCR Cloning Kit (Thermo Scientific).

All cloning was carried out using the GATEWAY™ system following the manufacturer’s protocols. The cDNAs of PpCHK1-3 were obtained [19,21]. For obtaining entry vectors, DNA sequences encoding sensory modules with adjacent transmembrane helices (SMs + TMs) from CHK genes *PpCHK3, PaCHK1* and *PaCHK2* were amplified using the respective primer pairs (Table S1). DNA sequences of SMs + TMs of putative receptors PpCHK4, SmCHK4a and SmCHK6 were synthesized by Evrogen (Moscow, Russia) and amplified in a two-step PCR (for primers see Table S1). The coding sequence was transferred via in vitro recombination to the destination vectors pK7WGF2 and pB7FWG2 as indicated in the text; in these constructs the *GFP* reporter gene was fused to *CHK* gene at its 5′ or 3′-terminus, respectively.

#### 4.3.2. Receptors Expression and CK Binding Assay

pK7WGF2 and pB7FWG2 constructs were transformed to *Agrobacterium tumefaciens* strain GV3101 and transiently expressed in *Nicotiana benthamiana* leaves. Isolation of leaf microsomes and their use for CK binding determination in radioligand assays with ^3^H-iP [87] were accomplished as described earlier [14]. Transient expression was assessed by means of immunoblotting of microsomal membranes with antibodies against GFP (Agrisera, AS15 2987) diluted 1:5000 as described in [15]. Background binding of ^3^H-iP by endogenous CK receptors of *N. benthamiana* was assessed in control probes using microsomes from untreated leaves (Figure 2). The difference between TB and NS, in other words, iP specific binding in control probes was negligible and statistically insignificant, its share in the measured specific binding being no more than 0.7–3.6%. This insignificant endogenous binding cannot alter in any way the observed ligand-binding properties of the receptors under study. A one-way ANOVA was used to compare the values (TB and NS for each receptor) resulting from the CK binding experiments. For all functional receptors, data on statistically extremely significant hormone specific binding (*p*-value ≤ 0.001) were obtained.

*K*_D_ for receptor interaction with various CKs was determined in competition experiments as described [88] according to Cheng-Prusoff calculation method [65]. The competition curves were generated using the simple ligand binding option of the SigmaPlot 12 program (Systat Software, San Jose, CA, USA). The goodness of fit for the non-linear regression model was estimated using the parameter R-squared (R^2^) which is a statistical measure of how close the data are to the fitted regression line. The R^2^ values for all tested CHKs were close to maximum value of 1.0. An alternative method for *K*_D_ calculation from competition binding assays was given in the Appendix A and used to perform independent determination of *K*_D_’s (Table S2).

## Figures and Tables

**Table 1:** Quantitation of the affinities of CHK receptors from early-divergent lineages for all essential CK versions (Cheng-Prusoff calculation method).

CK Version	*K*_D_ of CK-Receptor Complexes (nM ± SE) for:					
	PpCHK1	PpCHK2	SmCHK4a	SmCHK6	PaCHK1	PaCHK2
tZ	36.9 ± 2	18.1 ± 1.5	**7.99 ± 1.34**	**1.15 ± 0.20**	**7.57 ± 0.37**	**1.05 ± 0.09**
cZ	103 ± 15	34.2 ± 9.3	20.6 ± 2.6	16.6 ± 2.4	419 ± 56	17.9 ± 2.9
iP	**1.92 ± 0.06**	**1.78 ± 0.14**	**1.60 ± 0.13**	**1.70 ± 0.16**	**3.60 ± 0.17**	**2.20 ± 0.08**
DZ	507 ± 42	323 ± 97	54.8 ± 6.2	36.0 ± 1.6	377 ± 27	22.4 ± 2.9
BA	13.9 ± 0.1	10.7 ± 0.5	**6.91 ± 0.34**	**2.28 ± 0.70**	61.9 ± 1.3	13.5 ± 1.6
TD	23.8 ± 5.4	13.02 ± 0.2	**4.52 ± 0.62**	16.6 ± 4.7	**6.70 ± 0.21**	64.5 ± 6.4

Values less than 10 nM (attesting for high affinity) are highlighted in bold. Abbreviations: Pp, *Physcomitrium patens*; Sm, *Selaginella moellendorffii*; Pa, *Picea abies.*

## Data Availability

Not applicable.

## Supplementary Materials

The following are available online at https://www.mdpi.com/article/10.3390/ijms222313077/s1.

## Appendix A. A New Approach for KD Determination in Competition Experiments

In competition experiments on ligand binding to receptors, a binding curve of the labeled ligand is plotted in the presence of increasing concentrations of unlabeled ligands. In the standard approach, the apparent dissociation constants of the receptor and unlabeled ligands are calculated using the Cheng-Prusoff equation. This formula is derived from the expression for the saturation binding curve. It is important that this expression is valid only under the condition of high superiority in concentration of the ligand over the receptor. In this case, the initial equations are simplified up to the final expression. This leads to certain limitations in the design of the experiment, often making the technique costly and inconvenient to use. By contrast, the calculation methods based on the equation for the equilibrium dissociation constant are free from such restrictions.

The formulas for the dissociation constant of labeled and unlabeled hormones in competition experiments are as follows:

(A1) $$K_{D_{M}}=\frac{L_{M}*R}{LR_{M}};\;K_{D}=\frac{L*R}{LR}\;$$

Here *L*_M_ is the concentration of unbound labeled ligand, *LR_M_* is the concentration of bound labeled ligand, *L* is the concentration of unbound unlabeled ligand, *LR* is the concentration of bound unlabeled ligand, *R* is the concentration of free receptor. In turn, *R*, *L*_M_ and *L* can be expressed as follows:

(A2) $$R=R_{T}-LR_{M}-LR;\;L_{M}=L_{TM}-LR_{M};\;\;L=L_{T}-LR$$

Here *R_T_*, *L_TM_*, *L_T_* are the total concentrations of the receptor, labeled and unlabeled ligands prior to the initiation of the binding process. In a competition experiment, the three quantities are constants: *K*_DM_, *L_TM_* and *R_T_*. The first value is calculated in a separate saturation binding assay. The second is initially set by the researcher. The third value, knowing the two previous ones, can be calculated from the binding of the labeled ligand in the absence of an unlabeled ligand using the following equation obtained from Equations (A1) and (A2):

(A3) $$R_{T}\left(constant\right)=\frac{K_{D_{M}}*LR_{M}^{L_{T}=0}}{L_{TM}-LR_{M}^{L_{T}=0}}+LR_{M}^{L_{T}=0}$$

In the presence of an unlabeled ligand, taking into account Equations (A1) and (A2), we obtain the following equation for $K_{D_{M}}$:

(A4) $$K_{D_{M}}=\frac{\left(L_{TM}-LR_{M}\right)*\left(R_{T}-LR_{M}-LR\right)}{LR_{M}}$$

Hence, the concentration of the bound unlabeled ligand can be expressed as:

(A5) $$LR=\frac{K_{D_{M}}*LR_{M}}{LR_{M}-L_{TM}}-LR_{M}+R_{T}$$

Accordingly, taking into account Equations (A1) and (A2), *K*_D_ for an unlabeled ligand can be calculated using the equation:

(A6) $$K_{D}=\frac{\left(L_{T}-LR\right)*\left(R_{T}-LR_{M}-LR\right)}{LR}$$

*R_T_* and *LR* are defined in Equations (A3) and (A5), respectively. For greater accuracy, we calculated $K_{D}$ at several *L_T_*s and determined its average value. Results are shown in Table S2. Comparison of *K*_D_ values determined by two different calculation algorithms and displayed in Table 1 and Table S2 demonstrates reasonable consistency between these two datasets.
