# Supplementary material for: Cytokinin Perception in Ancient Plants beyond Angiospermae
Source: Int J Mol Sci. 2021 Dec 3;22(23):13077. doi: 10.3390/ijms222313077 (PMC8657898; doi:10.3390/ijms222313077)
Supplement: Supplementary file 1 [file ijms-22-13077-s001.zip › ijms-1484554-supplementary.pdf]

Supplementary data
to Lomin et al. Cytokinin Perception in Ancient Plants beyond Angiospermae

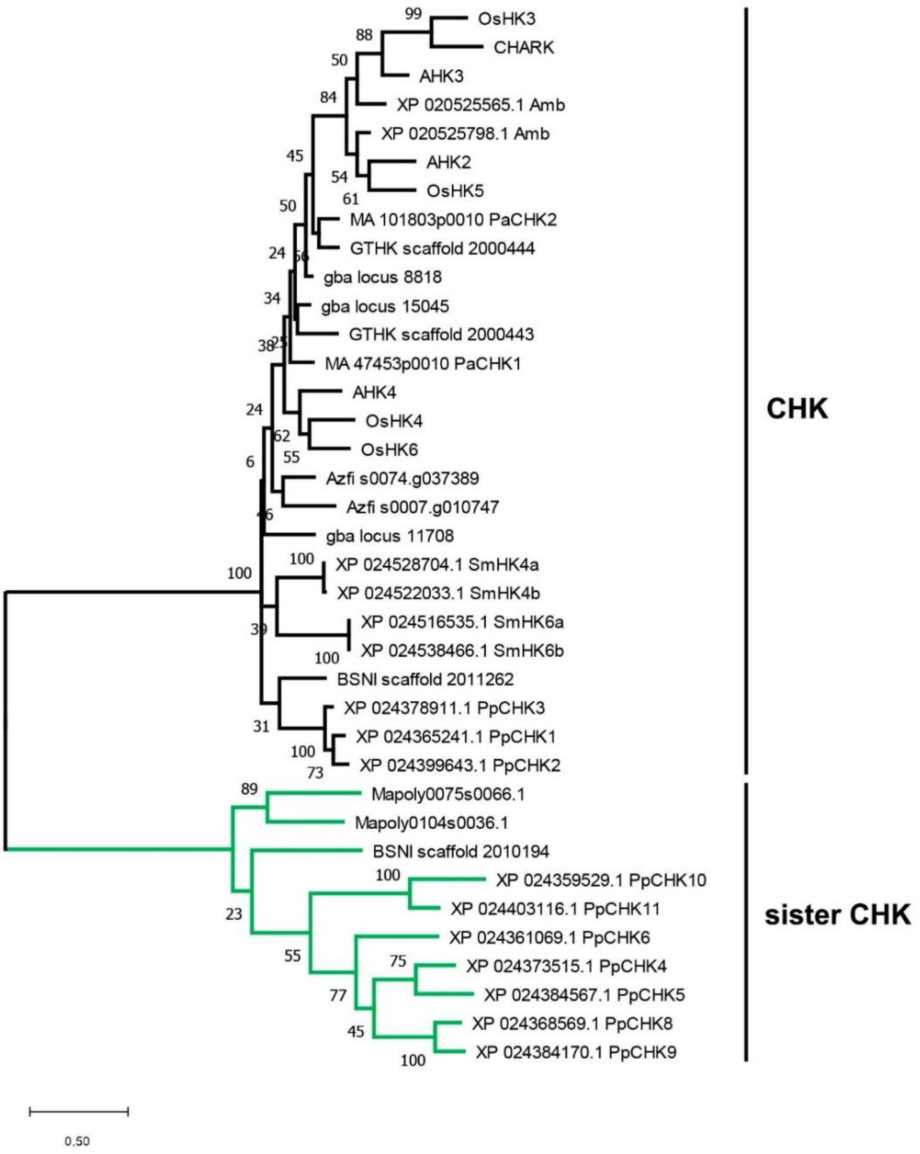

Figure S1. The CHK tree. The evolutionary history was inferred by using the Maximum Likelihood method and Poisson correction model (Zuckerkandl and Pauling, 1965). The tree with the highest log likelihood (-9637,20) is shown. The percentage of trees in which the associated taxa clustered together is shown next to the branches. Initial tree(s) for the heuristic search were obtained automatically by applying the Maximum Parsimony method. A discrete Gamma distribution was used to model evolutionary rate differences among sites (5 categories (+*G*, parameter = 1.2933)). The tree is drawn to scale, with branch lengths measured in the number of substitutions per site. This analysis involved 37 amino acid sequences. There were a total of 274 positions in the final dataset. Evolutionary analyses were conducted in MEGA11 (Tamura et al., 2021). Abbreviations in the tree correspond to histidine kinases from: AHK, *Arabidopsis thaliana* (dicot); OsHK/CHARK, *Oryza sativa* (monocot); Amb, *Amborella trichopoda* (basal angiosperm); gba, *Ginkgo biloba*; GTHK, *Gnetum montanum*; PaCHK, *Picea abies*; Azfi, *Azolla filiculoides*; Sm, *Selaginella moellendorffii*; PpCHK, *Physcomitrium patens*; Mapoly, *Marchantia polymorpha*; BSNI, *Anthoceros agrestis*. Except last three species, all other species belong to vascular plants, including representatives of Spermatophyta: divisions Magnoliophyta (*A. thaliana*, *O. sativa*, *A. trichopoda*), Ginkgophyta (*G. biloba*), Gnetophyta (*G. montanum*), Pinophyta (*P. abies*); and Polypodiophyta (*A. filiculoides*); representative of Lycopodiophyta (*S. moellendorffii*) is also included. Non-vascular Bryophytes are represented by divisions Bryophyta (*P. patens*), Marchantiophyta (*M. polymorpha*) and Anthocerotophyta (*A. agrestis*).

Zuckerkandl E. and Pauling L. (1965) Evolutionary divergence and convergence in proteins. Edited in *Evolving Genes and Proteins* by V. Bryson and H.J. Vogel, pp. 97-166. Academic Press, New York.

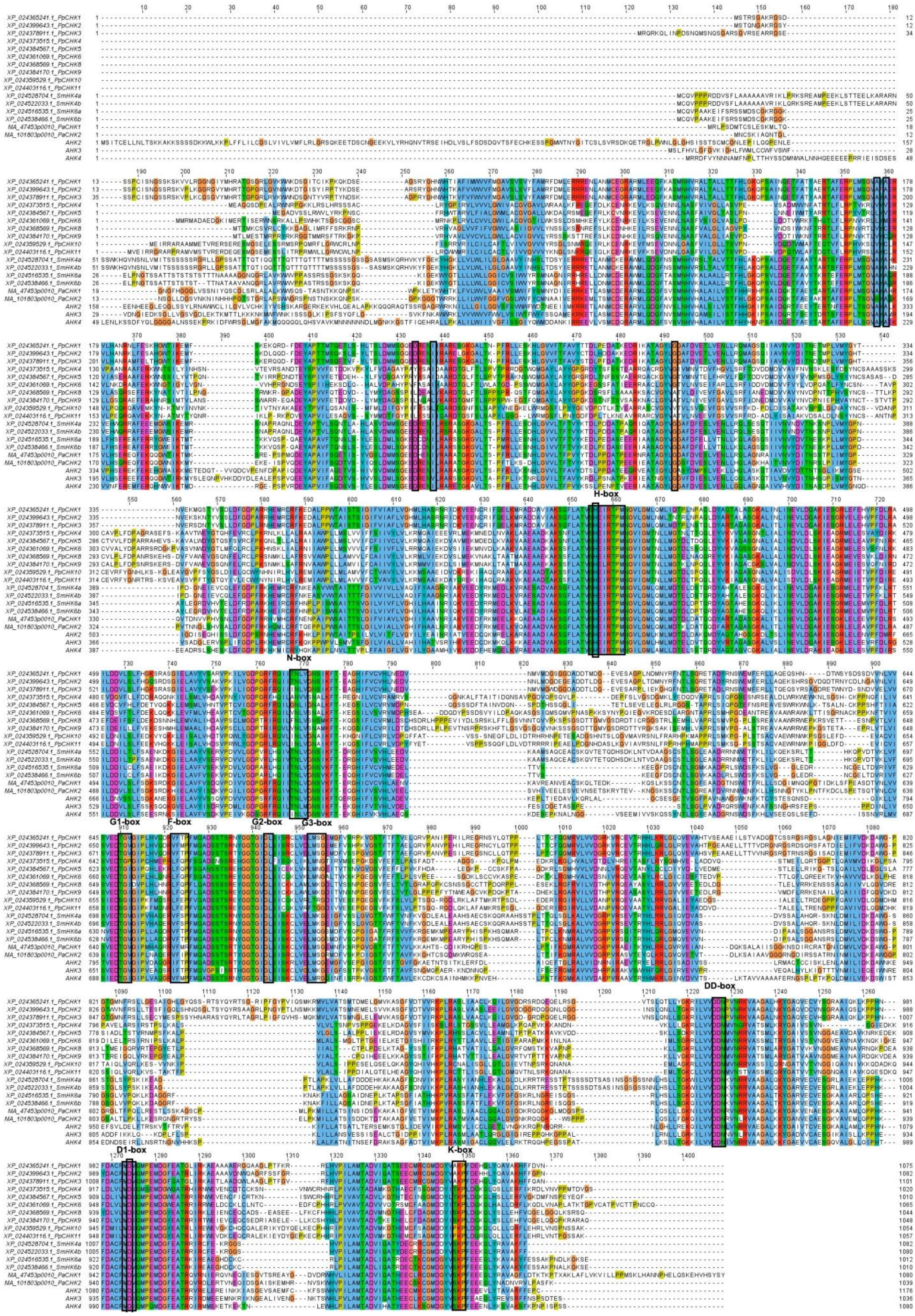

Figure S2. Alignment of cytokinin receptor protein sequences. Key amino acids and amino acid boxes required for ligand binding, histidine kinase activity, and phosphotransfer are highlighted.

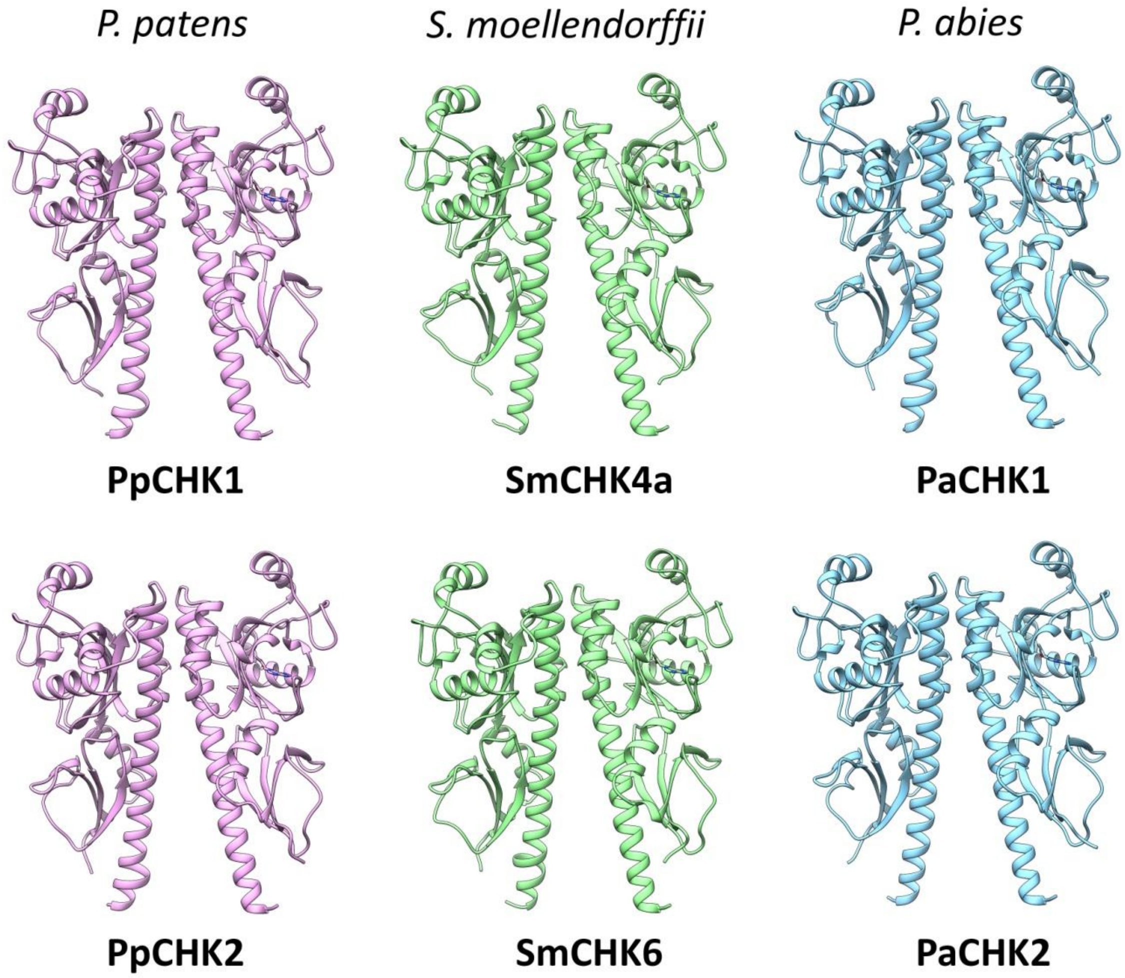

Figure S3. Homology models for the ligand binding sensory modules (in the form of homodimers) of genuine CHK receptors from *P. patens* (PpCHK1 and PpCHK2), *S. moellendorffii* (SmCHK4a and SmCHK6), and *P. abies* (PaCHK1 and PaCHK2). Each pair of CK receptors represents the CK perception apparatus of the respective species.

Table S1. Primers used in this study.

Primer name	Primer sequences (5' – 3')
PpCHK3SM_attB1	TACAAAAAAGCAGGCTTGATGGTTTTCGTTTTCTGGGTGG
PpCHK3SM_attB2	ACAAGAAAGCTGGGTAGAGATTTCGCTATGACAGC
PaCHK1SM_attB1	TACAAAAAAGCAGGCTTGATGTTGTTCGTTCTGTGG
PaCHK1SM_attB2	ACAAGAAAGCTGGGTAGTATATTATGTGACC
PaCHK2SM_ attB1	TACAAAAAAGCAGGCTTGATGTTGATTCTGTGGGTGTTTG
PaCHK2SM_ attB2	ACAAGAAAGCTGGGTATATGTGCCCAACTAACAGAGC
PpCHK4SM_attB1	TACAAAAAAGCAGGCTTGATGGTGCTAGTCGTCGTG
PpCHK4SM_attB2	ACAAGAAAGCTGGGTAGAGCACTAGGTAGAC
SmCHK4aSM_attB1	TACAAAAAAGCAGGCTTGATGTATCACGGCAAGCTG
SmCHK4aSM_attB2	ACAAGAAAGCTGGGTAGATTTGAGCCACCAG
SmCHK6SM_attB1	TACAAAAAAGCAGGCTTGATGTGGTATGGAACGCTAC
SmCHK6SM_attB2	ACAAGAAAGCTGGGTACAATATATGACCCACC
attB1	GGGGACAAGTTTGTACAAAAAAGCAGGCT
attB2	GGGGACCACTTTGTACAAGAAAGCTGGGTA

Table S2. Quantitation of the affinities of CHK receptors from early-diverging lineages for all essential CK versions (alternative calculation method)

CK version	K_D of CK-receptor complexes (nM±SE) for:					
	PpCHK1	PpCHK2	SmCHK4a	SmCHK6	PaCHK1	PaCHK2
*t*Z	24.8±2.9	12.4±1.5	8.0±2.9	3.1±0.8	5.9±1.0	1.3±0.4
*c*Z	94.4±26.3	48.6±9.7	20±4.9	39.0±5.1	285.6±69.4	22.7±3.2
iP	1.2±0.3	0.9±0.0	0.8±0.2	2.5±0.8	3.0±0.7	2.2±0.5
DZ	676±14.4	500.9±74.7	32.3±11.6	48.4±7.1	244.2±25.9	29.1±5.4
BA	12.8±3.0	14.9±1.2	3.2±0.8	2.9±0.4	38.4±2.6	12.3±3.9
TD	17.1±6.6.4	11.9±2.8	4.9±2.0	29.8±3.4	3.8±0.6	243.5±94.8

Values less than 10 nM (attesting for high affinity) were highlighted red.
Abbreviations: Pp, *Physcomitrium patens*; Sm, *Selaginella moellendorffii*; Pa, *Picea abies*
